# Zinc Oxide Nanoparticles and Zinc Sulfate Alleviate Boron Toxicity in Cotton (*Gossypium hirsutum* L.)

**DOI:** 10.3390/plants13091184

**Published:** 2024-04-24

**Authors:** Ismail Sanusi Nassarawa, Zhuolin Li, Longshuo Xue, Huazu Li, Uzair Muhammad, Shuijin Zhu, Jinhong Chen, Tianlun Zhao

**Affiliations:** 1 College of Agriculture and Biotechnology, Zhejiang University, Hangzhou 310058, China; 22016279@zju.edu.cn (I.S.N.); xuelongshuo@163.com (L.X.); 12016016@zju.edu.cn (H.L.); anasuzair80@gmail.com (U.M.); shjzhu@zju.edu.cn (S.Z.); 2 Hainan Institute, Zhejiang University, Sanya 572025, China; 22216197@zju.edu.cn

**Keywords:** cotton, ZnO nanoparticle, ZnSO_4_, boron toxicity

## Abstract

Boron toxicity significantly hinders the growth and development of cotton plants, therefore affecting the yield and quality of this important cash crop worldwide. Limited studies have explored the efficacy of ZnSO_4_ (zinc sulfate) and ZnO nanoparticles (NPs) in alleviating boron toxicity. Nanoparticles have emerged as a novel strategy to reduce abiotic stress directly. The precise mechanism underlying the alleviation of boron toxicity by ZnO NPs in cotton remains unclear. In this study, ZnO NPs demonstrated superior potential for alleviating boron toxicity compared to ZnSO_4_ in hydroponically cultivated cotton seedlings. Under boron stress, plants supplemented with ZnO NPs exhibited significant increases in total fresh weight (75.97%), root fresh weight (39.64%), and leaf fresh weight (69.91%). ZnO NPs positively affected photosynthetic parameters and SPAD values. ZnO NPs substantially reduced H_2_O_2_ (hydrogen peroxide) by 27.87% and 32.26%, MDA (malondialdehyde) by 27.01% and 34.26%, and O_2_^−^ (superoxide anion) by 41.64% and 48.70% after 24 and 72 h, respectively. The application of ZnO NPs increased the antioxidant activities of SOD (superoxide dismutase) by 82.09% and 76.52%, CAT (catalase) by 16.79% and 16.33%, and POD (peroxidase) by 23.77% and 21.66% after 24 and 72 h, respectively. ZnO NP and ZnSO_4_ application demonstrated remarkable efficiency in improving plant biomass, mineral nutrient content, and reducing boron levels in cotton seedlings under boron toxicity. A transcriptome analysis and corresponding verification revealed a significant up-regulation of genes encoding antioxidant enzymes, photosynthesis pathway, and ABC transporter genes with the application of ZnO NPs. These findings provide valuable insights for the mechanism of boron stress tolerance in cotton and provide a theoretical basis for applying ZnO NPs and ZnSO_4_ to reduce boron toxicity in cotton production.

## 1. Introduction

There has been recent controversy regarding the importance of boron (B) for plant growth, which has sparked an interesting discussion among “Boronists” [1]. The need for it in plant development was initially recognized in the early 20th century [2]. However, in dry and semiarid environments in particular, interactions between boron toxicity, salinity, and drought stressors frequently occur simultaneously, which might hinder boron influx by limiting water transpiration and uptake [3]. Being crucial for plant growth, it stands out in nature due to its uniqueness. Its clear distinction from other micronutrients lies in its low threshold between toxicity and deficiency [4,5]. It is one of the most important microelements for the growth of plants and is vital to many physiological functions [6].

Furthermore, boron can be found in high concentrations in groundwater and soil due to natural processes, or it can be added to the soil through fertilizer, irrigation water, and mining [7]. Most frequently, mature leaves will exhibit chlorosis, spotting, necrosis at the edges and tips, and consequently yield reduction which are the specific indications of boron toxicity [8]. In addition, boron poisoning sharply reduces photosynthetic activity, lowering chlorophyll levels and also altering the activity of antioxidant enzymes [9,10]. Zinc (Zn), a vital micronutrient, plays a pivotal role in regulating numerous physiological processes and mechanisms in plants. These include the biosynthesis of various hormones such as gibberellin, auxin, cytokinin, and abscisic acid, as well as the production of chlorophyll and the development of chloroplasts. Additionally, zinc influences the stability and structure of the protective cell membrane [11].

Zinc also plays an important role as a co-factor in several biocatalytic enzymes, such as hydrolases, ligases, isomerases, and transferases. These enzymes are involved in stomatal conductance regulation and ionic balance management. This control streamlines the path that vital micronutrients take as they pass through plant roots and are carried by the circulatory system to the aerial portions of plants [11]. Zn application to plants increased their overall development and reduced the stress on plants caused by boron toxicity, making the cotton plant withstandstress and encouraging root growth [12].

Recently, plant stress caused by both biotic and abiotic factors has been widely acknowledged to be mitigated by nanotechnology, which helps to maintain sustainable farming practices [13]. Applying nanoparticles (NPs) has been demonstrated to improve mineral accumulation at the subcellular level, which in turn has benefited plant growth [14]. Additionally, they strengthen the plant’s resistance to boron toxicity by controlling the activity of antioxidant enzymes, raising the rate of photosynthetic respiration, and ultimately helping to reduce excess boron in cotton plants [15]. Scientists are currently paying close attention to zinc oxide nanoparticles (ZnO NPs). They are essential and have a wide range of economic uses, which will eventually increase crop productivity and highlight the use of sustainable farming methods [16]. Furthermore, ZnO NPs are more economical, potentially non-toxic, ecologically friendly, and biocompatible due to their variety of applications [17]. Earlier studies have indicated that ZnO NPs serve as an economical and sustainable method that can improve soil fertility, reduce stress, and increase crop productivity [18].

Notably, cotton (*Gossypium hirsutum* L.) is among the principal suppliers of commercial cash crops that produce fiber worldwide [19]. The first step in growing commercially significant crops on contaminated land is developing novel, nanotechnology-based instruments. These instruments seek to increase agricultural yields by reducing boron toxicity and adjusting plant antioxidant defense systems. Currently, no thorough study has examined the potential effect of zinc sulfate and zinc oxide nanoparticles to reduce boron toxicity in cotton. As a result, our study focused on filling this research gap in the literature. Therefore, our study aimed to compare the effects of two different forms of zinc: zinc sulfate (ZnSO_4_) and ZnO NPs. The investigation also sought to explore the potential role of ZnO NPs in alleviating boron toxicity in cotton plants.

Generally speaking, cotton seedlings are less resistant to boron toxicity. We also looked at how ZnO NPs could improve cotton plants’ ability to withstand high boron concentrations. This was investigated in relation to altering the rate of photosynthesis, the ability to scavenge reactive oxygen species (ROS), and the biosynthesis of chlorophyll. As a result, our study illuminated the function of ZnO NPs in reducing elevated boron levels in cotton, providing insight into the corresponding physiological and molecular processes. This study highlighted potential resistance mechanisms and improvements in cotton’s response to boron stress, which may ultimately lead to increased crop output. It focused on new developments and insights into the usage of ZnO NPs in reducing boron stress in cotton plants.

## 2. Material and Methods

### 2.1. Nanoparticle Preparation

The zinc oxide nanoparticles (ZnO NPs), used in this study were purchased from Shanghai Chaowei Nanotechnology Co., Ltd., Shanghai, China. The manufacturer synthesized the ZnO NPs using a wet chemical method (sol-gel technique), which is a common and efficient approach for producing high-quality ZnO NPs [20,21]. The purchased ZnO NPs had a purity of 99% and an average diameter of 30 nm, as specified by the manufacturer. A stock solution with a concentration of 50 mg/L was prepared by dispersing an appropriate amount of ZnO NPs in deionized water. The suspension was then subjected to an ultrasonic cleaner for about 30 min to ensure proper dispersion and homogeneity of the nanoparticles [22]. The mixture became more homogeneous, and the particles appeared to be well dispersed in the solution. The stability of the ZnO NPs’ dispersion in the hydroponic solution was monitored over time to ensure that the plants received a consistent supply of nanoparticles throughout the experiment.

### 2.2. Plant Experiment Setup

The cotton seeds (*Gossypium hirsutum* L.) used in this research were obtained from the Zhejiang University Institute of Agriculture and Biotechnology. The selected seeds were pest-free, fully grown, and of excellent quality. They were immersed in distilled water at 25 °C for 24 h. The seeds were carefully sown in a 50-hole seedling tray with vermiculite to nutrient soil ratio of 1:1. Each hole received 1–2 cotton seeds, and the soil was covered. Adequate water was applied until reaching a moist state. Following this, the trays were transferred to a plant culture room for a germination and growth period lasting for seven days. The incubation conditions were maintained at a temperature of 25/22 °C (light/dark), a light intensity of 90–120 mol·m^−2^ s^−1^, a photoperiod of 14/10 h (light/dark), and a humidity of 65–70%. Once the two cotyledons of cotton seedlings had fully flattened, seedlings with the same basic growth characteristics (full cotyledons and consistent seedling height) were chosen. They were then moved to 1 L black hydroponic plastic buckets, with five plants per barrel. During the transfer process, damage to the root system was minimized, and ultrapure water was used for cultivation on the transplantation day to aid the cotton seedlings in adapting to the hydroponic growth environment. Subsequently, the ultrapure water was replaced with a 1/4 concentration of an improved Hoagland nutrient solution every 3–4 days. A preliminary screening experiment was conducted with different doses of ZnSO_4_·7H_2_O (Cas number: 7446-20-0) bought from Coolaber Co., Ltd. (Beijing, China), ZnO NPs (0–200 mg/L), and the boron in form of boric acid H_3_BO_3_ (Cas number: 10043-35-3) bought from Sinopharm Chemical Reagent Co., Ltd. The optimal concentrations were determined as 4 mg/L for ZnSO_4_, 50 mg/L for ZnO NPs, and 150 mg/L for boron. Based on the screening results, the hydroponic medium was prepared with an optimal dose of 4 mg/L of ZnSO_4_, 50 mg/L of ZnO NPs, and 150 mg/L of boron. Seedlings grown without additional ZnSO_4_, ZnO NPs, and boron served as the control, while the control medium contained a baseline level of boron (approximately 0.125 mg/L) from the ¼ concentration of improved Hoagland nutrient solution.

The study comprised six treatments, following three replications in a completely randomized design: CK, ZnSO_4_ (4 mg/L), ZnO NPs (50 mg/L), B (150 mg/L), B + Zn (150 mg/L + 4 mg/L), and B + ZnO NPs (150 mg/L + 50 mg/L). The experiments were arranged based on the number of treatments, and cotton plant samples were treated after the development of the 4th matured leaf. The samples were collected after 7 days of treatments. Physical and physiological characteristics of the phenotype as well as molecular determinations were measured and tested. Three replicates of cotton seedling root tissues were combined and kept at −80 °C after being directly immersed in liquid nitrogen. Measurements of seedling growth and biomass were conducted.

### 2.3. Measurement of Seedlings’ Growth and Biomass

After enduring a week of boron stress, cotton seedlings were harvested. Physiological data were recorded using an electronic balance, including the total fresh plant weight, shoot weight, root weight, and leaf weight. The cotton seedlings were then put in an envelope of paper and kept inside a furnace for 72 h at 65 °C until the desired weight was maintained. Subsequently, the dried weights of the cotton plant, roots, leaves, and shoots were measured and expressed in grams.

### 2.4. Photosynthetic Parameters

A portable photosynthesis equipment (Li-6400) infrared analyzer (Li-COR, Lincoln, NE, USA) was used to assess the photosynthetic properties, including transpiration rate (Tr), photosynthetic rate (Pn), stomatal conductance (Gs), and intercellular CO_2_ concentration (Ci). The measurement parameters, according to [23], were as follows: a photon flux density of 1000 μmol m^−2^ s^−1^, a CO_2_ concentration of 400 μmol^−1^, and a relative humidity of 60%. In each treatment, 15 seedlings were measured, and the measurements were taken at 11:00 a.m. on a sunny day. The third genuine cotton leaf was used for all of the measures mentioned above. The IRGA system was calibrated before the data were taken, and during the measurement period, the zero was changed about every half-hour. For sixty seconds, each leaf was contained in a gas exchange chamber. For every treatment, three records of every attribute assessed by the IRGA system were made.

### 2.5. Quantification of Chlorophyll Content

A solution with the following contents was made: 1.5 mL of acetone, 95% pure ethanol, and deionized water in the following ratios (45:45:10). According to the reported method [24], 0.1 g of fresh leaves were weighed, placed in a pre-cooled centrifuge tube with a few grinding beads at a ratio of 100:1, and quantified. The samples were held in complete darkness until the green hue was completely gone. Chlorophyll a and b absorbance measurements were made at 663 and 645 nm using a microplate reader (Bethon Instruments, Inc., Synergy H1, Woburn, MA, USA). A portable chlorophyll meter (SPAD-502 Plus) was used to measure the relative value of the chlorophyll content index (SPAD Value), with 15 plants evaluated for each treatment. Seven days following boron stress, one leaf per seedling was tested, and the average value was noted three times per leaf [25].

### 2.6. Estimation of Boron Content and Elemental Analysis

The HNO_3_ acid technique was used in the cotton sample digestion process. Following the classification of the dried materials into roots, shoots, and leaves, 0.1 g of the dried weight was quantified and put in a test tube holding 2 mL of HNO_3_. After leaving the mixture overnight, the samples were fully digested the next day using an Antoon Paar Microwave 3000 microwave digester (Graz, Austria), first for two hours at 80 °C and then for three more hours at 180 °C until the volume reached 1 mL. The digested solution was then filtered through filter paper, and the initial dilution was made by adding 9 mL of distilled water. Then, 8 mL of distilled water was added for the second dilution. The concentrations of zinc (Zn), magnesium (Mg), iron (Fe), potassium (K), and boron (B) were determined using an Inductively Coupled Plasma Optical Emission Spectroscope (ICP-OES, IRIS INTREPID II XSP) based on the methods outlined in reference [26].

### 2.7. Determination of Antioxidant Enzymes

Suzhou Coming Biotechnology Co., Ltd.’s (Suzhou, China) SOD, POD, and CAT enzyme kits were utilized to measure the antioxidant activity of these three compounds. The supernatant for the assays mentioned above was obtained by centrifuging 0.1× *g* of cotton root seedlings for 10 min at 4 °C at 8000× *g* after they had been homogenized in 1.5 mL of 50 mM Tris buffer (pH 7.5). Superoxide dismutase (SOD) activity was assessed by measuring the capacity of each unit to prevent a 50% photochemical reduction in nitro blue tetrazolium chloride (NBT), in accordance with [27]. The CAT activity was ascertained in accordance with [28], whereas the POD activity was assessed at 470 nm as a result of H_2_O_2_-oxidizing guaiacol [29].

### 2.8. Determination of MDA, H_2_O_2_, and O_2_^−^ Contents

After undergoing treatment for 24 h and 72 h, the cotton fresh root sample was retrieved for the assessment of superoxide anion (O_2_^−^), hydrogen peroxide (H_2_O_2_), and malondialdehyde (MDA) concentrations. The MDA accumulation was evaluated using the thiobarbituric acid technique, as detailed by [30], using commercial kits (Suzhou Comin Biotechnology Co., Ltd., Suzhou, China) at 532 nm and 600 nm. Protocols designed by [31] for commercial test kits (Suzhou Comin Biotechnology Co., Ltd.) were adhered to in order to measure the content of H_2_O_2_ and O_2_^−^.

### 2.9. Transcriptome Sequencing

Samples from each treatment were gathered for an RNA-Seq analysis after a 24 h boron treatment, during which ZnO NPs and ZnSO_4_ were applied to the boron toxicity treatment in an attempt to determine whether ZnO NPs were effective in reducing boron toxicity. To guarantee robustness, three biological duplicates of each treatment were carried out. In order to produce RNA samples, 1 g of RNA was used as the input material for each sample. An RNA Nano 6000 Assay Kit and a Bioanalyzer 2100 system (Agilent Tech., Santa Clara, CA, USA) were used to qualify and quantify RNA. The index-coded sample data were clustered using a cBot Cluster Generation System and a TruSeq PE Cluster Kit 3-cBot-HS (Illumina, San Diego, CA, USA) in accordance with the manufacturer’s instructions. The libraries were then sequenced using the Illumina Novaseq platform, yielding paired-end readings of 150 bp. Only high-quality paired-end clean readings were used for additional analyses. The clean readings were aligned with the *Gossypium hirsutum* cv. using the HISAT2.0.4 program [32]. Derived from the genetic information in the study by [33] on the TM genome, the analysis took into account both the gene’s length and the number of reads aligned to it, from which the anticipated number of fragments per kilobase of the exon model per million mapped fragments (FPKM) for each gene was calculated [34]. A differential expression analysis of the two treatments was performed using the DESeq2 R (1.20.0) program. DESeq2, which found differential expression in digital gene expression data using a model based on negative binomial distribution, provided statistical tools to control the false discovery rate; the obtained *p*-values were modified using the Benjamini and Hochberg methods. Differentially expressed genes were defined as those with an adjusted *p*-value (*p*-adj) < 0.05, as determined by DESeq2 [35].

### 2.10. Statistical Analysis

The significance difference between treatments was assessed by employing the SPSS 22.0 (IBM Corp., Armonk, NY, USA) and conducting a one-way analysis of variance (ANOVA) with a Duncan’s test at a 95% confidence interval using the mean values from three independent replicates. The graphical representation of the statistical study results was generated using TBtool (V 2.052) and Origin Pro 2021 (OriginLab Corporation, Northampton, MA, USA).

## 3. Results

### 3.1. Effect of ZnO NPs and ZnSO_4_ on the Fresh Weight and Dry Weight of Cotton Seedlings under Boron Toxicity

**Figure 1:**
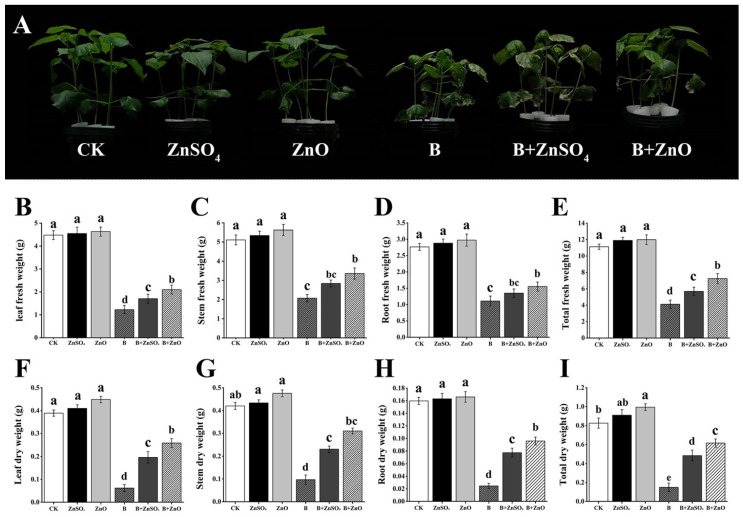
(**A**) Cotton seedlings at 7 days of treatment; (**B**) leaf fresh weight; (**C**) stem fresh weight; (**D**) root fresh weight; (**E**) total fresh weight; (**F**) leaf dry weight; (**G**) stem dry weight; (**H**) root dry weight; and (**I**) total dry weight. All the data are the mean of three replicates (n = 3), and the vertical bars demonstrate the standard deviation (SD). Different letters indicate significant differences among the treatments at (*p* ≤ 0.05). Control (CK), ZnSO_4_, ZnO NPs, boron (B), B + ZnSO_4_, B + ZnO NPs.

According to the current research, cotton plants exposed to 150 mg/L of boron showed poorer growth than the control group (CK) (Figure 1A). By comparing the growth of seedlings to different treatments, this conclusion was reached. In contrast to untreated seedlings (CK), cotton plants treated with ZnO NPs and ZnSO_4_ had shown a significant growth status. Interestingly, compared to the treatment with boron alone, the combined treatment of boron and ZnO NPs (B + ZnO NPs) showed improved outcomes, showing a considerable improvement. Growth reduction of 65.35% was observed in plants treated with 150 mg/L compared to the CK, according to quantitative measurements that took into account a number of parameters, including fresh total weight, leaf fresh weight, root fresh weight, stem fresh weight, and total dry weight, stem dry weight, leaf dry weight, and root dry weight. In contrast to B + ZnSO_4_, B + ZnO NPs caused an growth increase of 27.64% when compared to B + ZnSO_4_, indicating a significant difference. Interestingly, the application of B + ZnO NPs and B + ZnSO_4_ substantially alleviated boron-induced stress, reducing it to 75.97% and 37.86%, respectively, compared to boron treatment. Additionally, the positive impact of B + ZnO NPs was highlighted in the root fresh weight, which decreased by 61.45% with boron treatment compared with the control but increased by 39.64% and 21.62% when compared with B + ZnO NPs and B + ZnSO_4_, respectively, and increased by 14.81% when B + ZnO NPs were compared with B + ZnSO_4_. This research revealed that boron concentrations of 150 mg/L significantly decreased the dry weight of cotton plants. On the other hand, when B + ZnO NPs were introduced in comparison to B + ZnSO_4_, the total dry weight increased by 27.08%, 306.66%, and 220%, respectively, as compared to the boron treatment. Root dry weight, leaf dry weight, and stem dry weight all showed comparable patterns. Together, these results show that applying zinc oxide nanoparticles at the right concentration encouraged the roots, leaves, and shoots of cotton seedlings to grow and develop (Figure 1B–I).

### 3.2. RNA-Seq and Differentially Expressed Genes (DEGs) Analysis

In order to gain a deeper comprehension of how ZnO NPs and ZnSO_4_ mitigate boron toxicity in cotton, we ran an RNA-Seq and screened differentially expressed genes (DEGs) (Table 1). Following the establishment of the Pearson correlation coefficient between the biological replicates of our treatments, namely the CK, B (150 mg/L), B + ZnSO_4_ (150 + 4 mg/L), and B + ZnO (150 + 50 mg/L), we carried out a transcriptome analysis (Figure S3); the values of the Pearson correlation coefficient span from −1 to 1. Interestingly, for each of the four treatments, the current correlation coefficients for the three replicates are greater than 0.8. Consequently, test results obtained later can be considered trustworthy, and each material’s biological repeatability is strong.

A total of 22,739 differentially expressed genes (DEGs) were identified through screening across all materials; all DEGs were significanct with a *p*-adj value of 0.05. The up-regulated genes were within significance with a log2 fold change value of more than 1 whereas the down genes had a less than −1 log2 fold change value with a *p*-adj value of less than 0.05, encompassing B vs. CK (15,509), B + ZnSO_4_ vs. B (1086), B + ZnO vs. B (4000), and B + ZnO vs. B + ZnSO_4_ (2144). The number of DEGs in B vs. CK (15,509) was nearly fifteen times greater than that in B + ZnSO_4_ vs. B (1086), and approximately three times higher than B + ZnO vs. B (4000). Notably, the number of DEGs in B + ZnO vs. B (4000) surpassed that in B + ZnSO_4_ vs. B (1086) by almost four times, suggesting the significant role of ZnO NPs in alleviating the toxicity of boron treatment compared to the application of ZnSO_4_ in plant development or growth. Subsequently, GO and KEGG enrichment analysis were conducted on up-regulated genes. The GO enrichment analysis of up-regulated genes in the B + ZnO vs. B comparison revealed a significant enrichment of 79 GO terms. Noteworthy enriched terms included “sulfate transport”, “extracellular region”, “vitamin binding”, and others. Furthermore, the KEGG enrichment analysis of up-regulated genes in the B + ZnO vs. B comparison identified enrichment pathways, such as “Photosynthesis”, “ABC transpoters”, and “Glutathione metabolism” (Figure S2).

Furthermore, gene ontology (GO) and Kyoto Encyclopedia of Genes and Genomes (KEGG) pathway enrichment analysis were performed to obtain a deeper understanding of the roles of the differentially expressed genes (DEGs) associated with the B vs. CK, B + Zn vs. B, B + ZnO vs. B, and B + ZnO vs. B + ZnSO_4_ comparison groups. The top 30 terms that produced enrichment findings from the GO enrichment analysis of DEGs are shown in Figure S1. There were differences between the four comparison groups in the quantity of genes linked to biological processes, molecular functions, and cell components. In the B vs. CK, B + ZnSO_4_ vs. B, B + ZnO vs. B, and B + ZnO vs. B + ZnSO_4_ comparison groups, the DEGs showed enrichment in 10 cellular components, 10 biological processes, and 10 molecular functions (Figure S1).

### 3.3. ZnO NPs and ZnSO_4_ Application Enhances Photosynthetic Parameters and Chlorophyll Contents of Cotton Seedlings under Boron Toxicity

**Figure 2:**
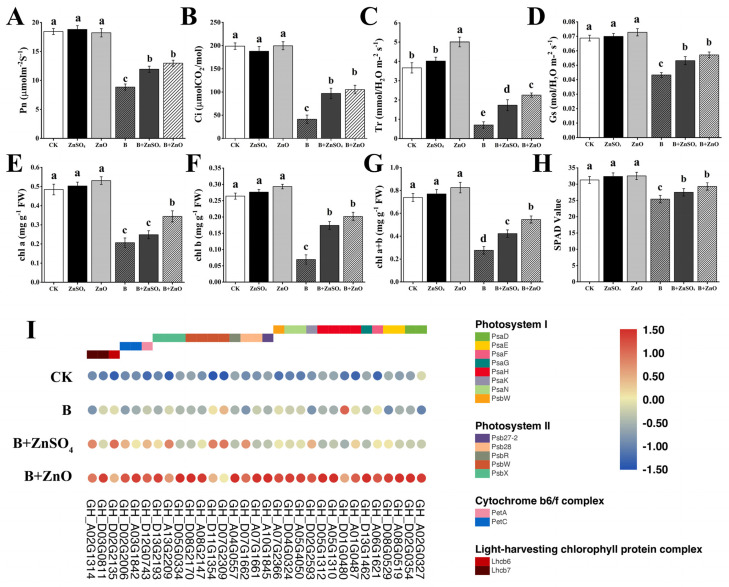
The effect of ZnO NPs and ZnSO_4_ under boron toxicity on photosynthetic parameters and chlorophyll contents of cotton 7 days after treatment. All the data are the mean of three replicates (n = 3) and the vertical bars demonstrate the standard deviation (SD). Different letters indicate significant differences among the treatments at (*p* ≤ 0.05). (**A**) Photosynthetic rate (Pn); (**B**) intercellular CO_2_ concentration (Ci); (**C**) transpiration rate (Tr); (**D**) stomatal conductance (Gs); (**E**) chlorophyll a (Chla); (**F**) chlorophyll b (Chlb); (**G**) chlorophyll a + b (Chla + Chlb); (**H**) SPAD value; (**I**) the expression of genes encoding a photosynthesis pathway in the B + ZnO vs. B comparison. The gene expression in the figure is indicated by the fragments per kilobase of the exon model per million mapped fragments (FPKM) value log2 logarithm going through the row scale.

In the comparison between B + ZnO vs. B, we concentrated on screening genes linked to photosynthesis after the enrichment of KEGG keywords and pathways related to photosynthesis. After that, we looked at these genes’ transcriptional levels (Figure 2A). Most of the genes associated with photosynthesis were significantly altered, according to our findings; these included those involved in photosystem I (*PsaD*, *PsaE*, *PsaF*, *PsaG*, *PsaH*, *PsaK*, *PsaN*, and *PsaW*), photosystem II (*PSB27-2*, *PSB28*, *PSBR*, *PSBW*, and *PSBX*), the chrome b6/f complex (*PetA* and *PetC*), and the light-harvesting chlorophyll protein complex (*Lhcb6* and *Lhcb7*) (see Figure 2I). Significantly more than when compared to a single boron treatment, all of these genes showed considerable up-regulation after being treated with ZnO NPs under boron toxicity (Figure 2I). This suggests that in response to boron toxicity, ZnO NPs promoted a rise in the transcriptional levels of genes linked to photosynthesis, quickly increasing the rate of photosynthesis. The harmful effects of the boron treatment on cotton plants were therefore lessened as a result.

Boron toxicity significantly inhibited photosynthesis in cotton (Figure 2). When cotton seedlings were subjected to boron toxicity, the photosynthetic rate (Figure 2A) for B + ZnO NPs increased by 8.80% compared to B + ZnSO_4_. Additionally, B + ZnO NPs increased the photosynthetic rate by 46.50% compared with boron. In Figure 2B, the CO_2_ concentration (Ci) showed a decrease of boron (B) content by 79.22% compared to the CK. When compared to boron (B), B + ZnO NPs considerably increased this concentration by 134.73% (Figure 2B). Furthermore, Figure 2C shows a 30.64% increase in the transpiration rate (Tr) for B + ZnO relative to B + ZnSO_4_. This shows that while treating boron toxicity, using ZnO may improve leaf photosynthetic efficiency. Furthermore, when comparing boron to the CK, the stomatal conductance (Gs) dropped by 37.09%. On the other hand, Stomatal conductance increased by 32.09% when B + ZnO was contrasted with the boron treatment (Figure 2D).

The amount of chlorophyll content in the cotton leaf, represented as total chlorophyll, chlorophyll a, and chlorophyll b, and the SPAD value were shown in (Figure 2E–H). Chlorophyll content was significantly impacted by boron toxicity. Chlorophyll a, chlorophyll b, total chlorophyll, and SPAD values dropped following the 150 mg/L treatment in comparison to plants cultivated without boron stress. However, compared to the CK, plants receiving ZnO NP feed demonstrated a positive effect on chlorophyll concentrations. The plants treated with ZnO NPs and ZnSO_4_ in combination with boron showed a significant increase in chlorophyll content when compared with their corresponding plant under boron toxicity. The results revealed that the application of 50 mg/L of ZnO NPs in combination with boron enhanced chl a, chl b, chl a + b, and SPAD values. Specifically, compared with the CK, Boron (B) was showed a significant decrease in levels of 57.14%, 73.70%, 62.60% and 18.85% respectively, while an increase of 66.52%, 190.25%, 97.63%, and 15.35% was discovered when B + ZnO NPs were compared with the boron (B). Moreover, when B + ZnO NPs were compared with B + ZnSO_4_, it showed an increase in levels of 38.63%, 15.75%, 29.21%, and 6.55% (Figure 2E–H).

### 3.4. ZnO NPs and ZnSO_4_ Application Regulates Mineral Nutrients and Reduced Boron Contents in a Boron-Stressed Cotton Seedlings

**Figure 3:**
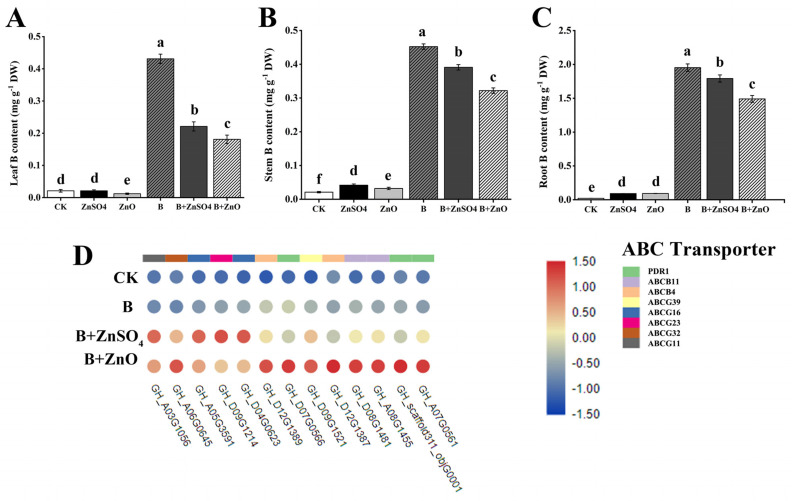
The boron concentration in the leaf, stem, and root parts were measured. (**A**) Leaf boron content; (**B**) stem boron content; (**C**) root boron content; (**D**) the expression of genes encoding the ABC transporter in the B + ZnO vs. B comparison. All the data are the mean of three replicates (n = 3), and the vertical bars demonstrate the standard deviation (SD). Different letters indicate significant differences among the treatments at (*p* ≤ 0.05). (**A**) Root B content; (**B**) stem B contents; (**C**) leaf B content.

In the present study, our findings showed that boron toxicity leads to the reduced accumulation of essential elements in the leaf, stem, and root of cotton seedlings (Table 2). In order to ascertain the impact of ZnO NPs on the elemental concentration of 150 mg/L boron-treated seedlings, we identified the ABC transporters gene in KEGG DEGs in cotton because these transporters require energy in order to transport ions that are necessary for plant growth and development, including their roles in the formation of protective layers and the transportation of phytohormones. Additionally, we identified the genes that were expressed in the B + ZnO vs. B comparison. Particularly, “ABCB11”, “ABCG32”, and “ABCG23” are involved in the ions that the plant needs. (Figure 3D). When cotton seedlings were exposed to high concentrations of boron, the boron content in the roots was higher than that in the above-ground parts of the plant, as well as the remaining elements, and also when the cotton seedlings were treated with B + ZnO NPs and B + ZnSO_4_, the boron concentration in the cotton seedlings’ tissues were decreased significantly, specifically with B + ZnO NPs. When boron treatment was compared with the CK, it recorded a significant decrease in the Mg of the root, stem, and leaf by 58.60%, 66.51%, and 16.91%, while the K of the root, stem, and leaf also decreased by 44.36%, 58.59%, and 26.91%, respectively. Additionally, Fe decreased in the stem and leaf by 57.14% and 60.00%, while Zn also decreased in the root and leaf by 14.55% and 71.43%, respectively. Our findings demonstrated that the cotton plant’s stem and root tissues experience a greater reduction in mineral nutrients when exposed to boron. ZnO NPs and ZnSO_4_ supplies, however, considerably lessened the reduction in element concentrations in the boron-stressed plants. For the boron content, B + ZnO NPs and B + ZnSO_4_ treatment decreased this by 23.59% and 8.20%, 28.89% and 15.38%, and 58.14% and 48.84% in the root, stem, and leaf, respectively (Figure 3A–C). Moreover, the expression of ABC transporters genes including *PDR1*, *ABCB11*, *ABCB4*, *ABCG39*, *ABCG16*, *ABCG23*, *ABCG32*, and *ABCG11*, was up-regulated, which confirmed that ZnO NPs and ZnSO_4_ decrease boron accumulation by regulating the ABC transporter. In conclusion, our results suggest the use of ZnO NPs as a possible technique to improve stress tolerance in cotton seedlings under boron toxicity.

### 3.5. ZnO NPs and ZnSO_4_ Application Enhances the Antioxidant Activity and Reduces Oxidative Stress in the Root of the Cotton Seedlings under Boron Toxicity

**Figure 4:**
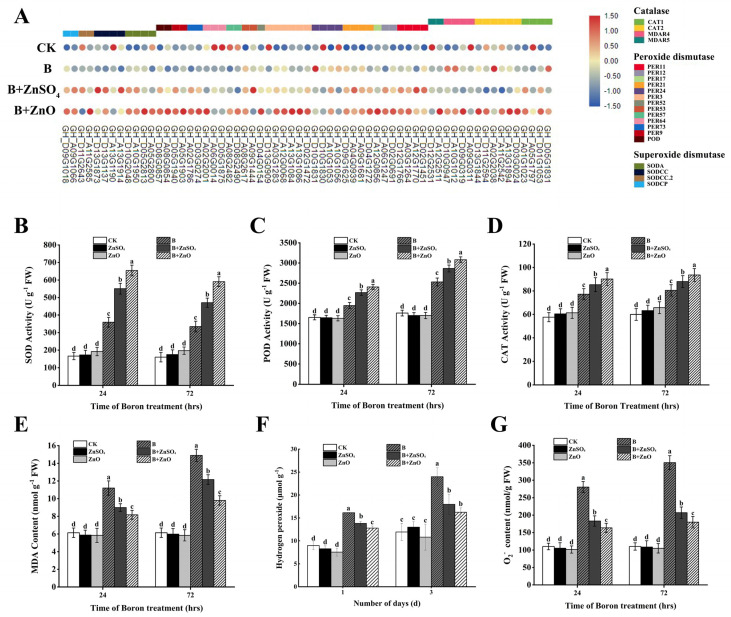
The effects of ZnO NPs and ZnSO_4_ on antioxidant activity and oxidative stress. (**A**) The gene expression of antioxidant enzymes in the B + ZnO vs. B comparison is illustrated through a heat map, with values estimated by calculating the fragments per kilobase of exon per million mapped fragments (FPKM) value; (**B**) superoxide dismutase (SOD); (**C**) peroxide dismutase (POD); (**D**) catalase (CAT); (**E**) malondialdehyde (MDA); (**F**) hydrogen peroxide (H_2_O_2_); and (**G**) superoxide anion content (O_2_^−^). The presented data represent the mean of three replicates (n = 3), and the vertical bars indicate the standard deviation (SD). Distinct letters signify a significant difference among the treatments at (*p* ≤ 0.05).

According to our findings, the use of ZnO NPs and ZnSO_4_ in the presence of boron toxicity resulted in the activation of numerous genes associated with the antioxidant system (Figure 4A). Specifically, when applying 50 mg/L of ZnO NPs under conditions of boron toxicity, genes such as *PER52*, *PER11*, *POXN1*, *PER73*, and *PER64* (encoding peroxide dismutase), *SODA*, *SOD2*, *SODCC*, and *SODCP* (encoding superoxide dismutase), and some catalase-encoding genes *CAT1*, *CAT2*, and *MDAR4* were significantly up-regulated (Figure 4A). These results highlight how antioxidant enzyme activities were modulated, indicating that ZnO NPs may enhance the ability to counteract reactive oxygen species (ROS). To identify their pivotal roles in alleviating boron-induced redox toxicity, the activities of antioxidant enzymes (SOD, POD, and CAT) were examined in cotton plants. The activity of these enzymes significantly increased at 24 and 72 h after treatment with 150 mg/L of boric acid compared to the control (Figure 4B–D). When comparing cotton seedlings treated with boron toxicity to those treated with B + ZnO (150 + 50 mg/L), the levels of SOD, POD, and CAT increased by 82.09% and 76.52%, 23.77%, and 21.66%, and 16.79% and 16.33%, respectively, after 24 and 72 h (Figure 4B–D). Our research indicates that applying a 50 mg/L ZnO NP solution to cotton plants exposed to 150 mg/L of boron may assist the plants by enhancing the activities of antioxidant enzymes and reducing ROS levels, thereby minimizing redox toxicity.

The MDA content of the cotton seedlings was assessed. When B treatment was compared with the control, our results clearly showed that the level of MDA content increased to 82.48% and 142.81% after 24 and 72 h, respectively. However, after 24 h and 72 h, comparing the treatment of B + ZnO NPs with plants treated with boron, there was a substantial drop in MDA content by 27.01% and 34.26% after 24 h and 72 h, respectively. (Figure 4E). This shows that ZnO NPs can lower the build-up of MDA content. Additionally, for hydrogen peroxide (H_2_O_2_), boron exhibited a substantial rise in H_2_O_2_ content by 52.00% and 100.96% after 24 h and 72 h, respectively, when compared with the control. Furthermore, a reduction in H_2_O_2_ content by 27.87% and 32.26% was noted after 24 and 72 h respectively, when B + ZnO NPs was compared with boron treatment. (Figure 4F). Thus, a measurement of the superoxide anion (O_2_^−^) content build-up was made. After 24 and 72 h, respectively, O_2_^−^ increased considerably by 154.13% and 217.78% when boron treatments were compared with the CK. When B + ZnO NPs were compared to boron (B), there was a substantial drop in O_2_^−^ by 41.64% and 48.71% after 24 h and 72 h respectively,. Also a decreased in O_2_^−^ by 10.66% and 13.14% was observed after 24 h and 72 h, respectively, when B + ZnO NPs was compared with B + ZnSO_4_ (Figure 4G).

## 4. Discussion

**Figure 5:**
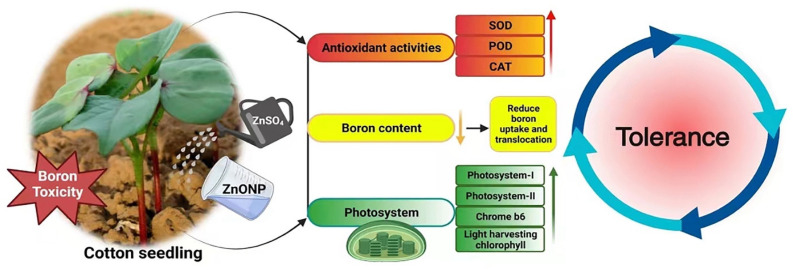
The overall mechanism of ZnO NPs and ZnSO_4_ in alleviating boron toxicity in cotton.

The primary objective of this study was to examine whether ZnO NPs and ZnSO_4_ could serve as an effective and environmentally friendly amendment to mitigate oxidative stress induced by boron toxicity; if yes, then which one is more effective? This investigation aimed to explore the physiological, chemical, and gene expression changes in cotton seedlings (*Gossypium hirsutum* L.). While nanoparticles are recognized for their environmental cleanup roles, the precise mechanism through which nanoparticles enhance plant growth and development remains not fully understood [36]. It is theorized that nanoparticles, owing to their large volume and surface area, can engage with cellular biomolecules, thereby initiating diverse biochemical pathways [37]. Recent studies have highlighted the efficacy of nanotechnology in agriculture, particularly regarding abiotic stress tolerance in various plant species [38]. Zinc nanoparticles (NPs) release zinc, a micronutrient crucial for regulating plant development [39]. The role of zinc in auxin biosynthesis, known for its involvement in cell division and cell expansion, has been well documented, contributing to overall plant growth [40]. However, there is not much room for error between a boron deficiency and toxicity [41]. Boron toxicity significantly hampers the growth and development of plants, with cotton, a key cash crop known for its fibers, being particularly susceptible to high levels of boron, thereby impacting its quality and yield. Despite its economic importance, cotton faces challenges due to elevated boron concentrations in the soil, negatively affecting both yield and quality. Zinc oxide nanoparticles (ZnO NPs), a novel nanotechnology, have demonstrated potential in alleviating abiotic stress in cotton plants. However, the precise mechanism by which ZnO NPs and ZnSO_4_ alleviate boron toxicity in cotton remains unclear. Our research revealed that ZnO NPs effectively mitigated boron toxicity, surpassing the efficacy of ZnSO_4_ (Figure 5). The results of this investigation showed that boron toxicity inhibited plant growth, resulting in shorter root and shoot lengths (Figure 1). On the other hand, the application of ZnO NPs led to a decrease in boron build-up in both the above-ground and root sections, as well as a notable rise in the weight of the roots, leaf, and shoots. As a buffer against boron toxicity, roots under ZnO NP treatment had a higher boron level than the above-ground portions; this lessened the detrimental effects on plant growth (Figure 3). Significantly, ZnO NP supplementation increased seedling resistance to boron toxicity by increasing the photosynthetic parameters (Figure 2) and growth rate (Figure 1) activating the photosynthetic pathway (Figure 2I) and elevating chlorophyll (Figure 2) Moreover, ZnO NPs were discovered to reduce boron toxicity by increasing the expression of genes linked to the ABC transporter pathway and the antioxidant system. Thus, the research indicates that boron toxicity in cotton plants may be mitigated by using ZnO NPs. Excessive boron led to decreased antioxidant enzyme activity; under boron toxicity, ZnO NPs were applied, and increased the activities of SOD, POD, and CAT (Figure 4A–D) while decreasing the amounts of H_2_O_2_, MDA, and O_2_^−^ in the roots (Figure 4E–G). This suggests that ZnO NP treatment enhanced antioxidant enzyme activity and decreased reactive oxygen species (ROS) levels under boron toxicity. ROS can function as signaling molecules and initiate several detoxification processes; this is in correspondence with [42]. When treating boron toxicity, the application of ZnO NPs may alter the genes linked to phytohormone control, supporting plant growth [43]. Particularly important in controlling plant growth in response to boron toxicity are plant hormones like salicylic acid [44], abscisic acid [45], auxin [46], cytokinins [47], ethylene [48], gibberellin acids [49], and jasmonic acid [50]. The application of ZnO NPs resulted in an elevation of leaf zinc content and a decrease in boron content, thereby enhancing chlorophyll levels and photosynthetic parameters in the presence of boron toxicity. Comparable positive outcomes of ZnO NPs were documented in cotton plants facing cadmium toxicity [51]. Adding ZnSO_4_ and ZnO NPs into the hydroponic system decreased the amount of boron in the leaves and shoots while simultaneously increasing the concentration of zinc in the shoot tissues (Table 2). Likewise, the reduction in boron toxicity through zinc application was noted in lemons [52] and grapefruit [53]. The elevated boron concentration in plants without zinc treatment may result from increased boron transport from roots to shoots. This suggestion aligns with [54]’s observation that the roots of zinc-sufficient plants seem to effectively limit boron accumulation in the above-ground parts, even though the precise mechanism for this restriction remains unknown. The compromised membrane integrity under ZnO NPs can impact the uptake and accumulation of boron at toxic levels in plants. The inhibitory impact of zinc on boron absorption suggests that zinc serves as a protective mechanism against the excessive uptake of boron, as noted by [55]. The addition of ZnSO_4_ and ZnO NPs mitigated the suppressive effect of high boron levels on plant dry weight and growth parameters (Figure 1A–I). Similar observations of zinc diminishing the toxic effects of boron on plant growth were reported in wheat [56], sour oranges [57], tomatoes (Lycopersicon esculentum) [58], mustard [59], and corn [54]. Under boron stress, water use efficiency experienced a decrease. Zinc might contribute to stomatal regulation by preserving membrane integrity, as suggested by [60]. However, a more in-depth investigation is needed to understand the specific role of ZnO NPs in stomatal regulation. Typically, when boron stress induces stomatal closure, leaf water use efficiency remains relatively constant, given that the reduction in transpiration is slightly less than the reduction in net photosynthesis (Figure 2A–D). The results of our investigation showed that cotton plants under boron toxicity had significantly lower levels of mineral nutrient components, such as K, Mg, Fe, and Zn, in the root, shoot, and leaf. Prior studies on rice have revealed similar findings [51]. Several causes could be responsible for the lack of substantial variations in Zn content in the stem and Fe content in the roots between the boron toxicity treatments and the control group. One hypothesis is that, in situations where boron toxicity is present, distinct mechanisms may control the absorption and translocation of these micronutrients. Numerous parameters, such as pH, redox potential, and the presence of other nutrients, have been found to affect the absorption and transportation of Fe in plants [61,62]. It is possible that in our investigation, the boron toxicity treatment had no discernible effect on these root zone parameters, leading to comparable Fe uptake to that of the control. Similar to this, the plant’s capacity to preserve Zn homeostasis in this tissue may account for the stem’s unaltered Zn level in the presence of boron toxicity. In order to prevent toxicity or deficiencies, plants have developed systems to control the absorption, translocation, and distribution of zinc [63]. Even in times of stress, the stem may give priority to maintaining zinc levels because it is an essential organ for nutrient delivery. Moreover, there is still much to learn about the intricate interactions that boron has with other nutrients like Fe and Zn. There has been research that shows boron and other minerals to have antagonistic effects [54,56], while others have observed synergistic or neutral interactions [64]. The specific response may depend on factors such as plant species, growth stage, and the severity of boron toxicity. Further research is needed to clarify the precise mechanisms governing the uptake and distribution of Fe and Zn in cotton plants under boron toxicity conditions. Our findings highlight the need for a more comprehensive understanding of nutrient interactions and their impact on plant growth and development.

## 5. Conclusions

In conclusion, our study demonstrated that the application of ZnO NPs and ZnSO_4_ effectively alleviated boron toxicity in cotton seedlings, with ZnO NPs showing superior potential compared to ZnSO_4_. The positive effects of ZnO NPs were evident in various physiological and biochemical parameters, such as increased photosynthesis rates, chlorophyll content, and ROS scavenging ability, as well as reduced boron content and enhanced antioxidant activity under boron toxicity conditions. The underlying mechanisms of ZnO NPs in alleviating boron toxicity were explored through a transcriptome analysis and corresponding verification, which revealed the significant up-regulation of genes encoding antioxidant enzymes, the photosynthesis pathway, and ABC transporter genes. These findings provide valuable insights into the molecular basis of ZnO NP-mediated boron stress tolerance in cotton plants.

In summary, this study has provided promising results on the use of ZnO NPs and ZnSO_4_ for alleviating boron toxicity in cotton; future studies should focus on determining the most effective concentration ranges, application methods, and timing of ZnO NP and ZnSO_4_ treatment for maximum benefits. Additionally, investigating the long-term effects of these treatments on soil health, crop yield, and quality is crucial for developing sustainable agricultural practices in boron-contaminated soils, by continuing to advance our understanding of these treatments and their underlying mechanisms.

## Figures and Tables

**Table 1:** DEGs in comparison groups.

Comparison Group	Total DEGs	Up-Regulated Genes	Down-Regulated Genes
B vs. CK	15,509	6261	9248
B + ZnSO_4_ vs. B	1086	915	171
B + ZnO vs. B	4000	2408	1592
B + ZnO vs. B + ZnSO_4_	2144	1022	1122

Total DEGs: Total number of differentially expressed genes; up: the number of up-regulated DEGs; down: the number of down-regulated DEGs.

**Table 2:** Effect of application of ZnO NPs on elemental concentration (mg/g) in root, stem, and leaf of cotton seedlings under boron toxicity.

Treatments	Root				Stem				Leaf			
	Mg	K	Fe	Zn	Mg	K	Fe	Zn	Mg	K	Fe	Zn
CK	4.30 a	36.34 b	0.60 b	0.55 d	2.18 ab	109.93 a	0.14 b	0.09 c	6.09 a	48.08 a	0.15 a	0.07 c
ZnSO_4_	1.89 b	51.78 a	1.38 a	3.16 c	2.61 a	55.58 b	0.09 b	0.50 a	6.08 a	46.71 a	0.09 b	0.46 a
ZnO	1.26 b	18.23 c	0.76 b	13.95 a	1.15 c	30.79 b	0.25 a	0.55 b	1.74 c	14.15 d	0.05 b	0.15 b
B	1.78 b	20.22 c	0.60 b	0.47 d	0.73 c	45.52 b	0.06 c	0.09 c	5.06 a	35.14 ab	0.06 b	0.12 b
B + ZnSO_4_	2.06 b	54.31 a	1.35 a	3.21 c	1.77 bc	57.24 b	0.03 c	0.45 a	5.06 a	40.15 a	0.08 b	0.18 b
B + ZnO	1.46 b	13.72 d	0.47 c	10.13 b	0.56 d	52.31 b	0.03 c	0.58 a	3.00 b	22.45 c	0.05 b	0.17 b

Data were the means of three independent replications for cotton seedlings; different letters represent significant differences between the treatments (*p* ≤ 0.05).

## Data Availability

All data are contained within the article.

## Supplementary Materials

The following supporting information can be downloaded at: https://www.mdpi.com/article/10.3390/plants13091184/s1, Figure S1: The top 30 of significantly upregulated GO enrichment analysis of differentially expressed genes in (A) B vs. CK (B) B + ZnSO_4_ vs. B (C) B + ZnO vs. B (D) B + ZnO vs. B + ZnSO_4_. Figure S2: The upregulated KEGG pathway enrichment analysis of differentially expressed genes in (A) B vs. CK (B) B + ZnSO_4_ vs. B (C) B + ZnO vs. B (D) B + ZnO vs. B + ZnSO_4_. Figure S3: Heat map of inter-sample correlations. The horizontal and vertical coordinates in the figure are the squares of the correlation coefficients of each sample.
