# Supplementary material for: Zinc Oxide Nanoparticles and Zinc Sulfate Alleviate Boron Toxicity in Cotton (Gossypium hirsutum L.)
Source: Plants (Basel). 2024 Apr 24;13(9):1184. doi: 10.3390/plants13091184 (PMC11085453; doi:10.3390/plants13091184)
Supplement: Supplementary file 1 [file plants-13-01184-s001.zip › plants-2926020-supplementary.pdf]

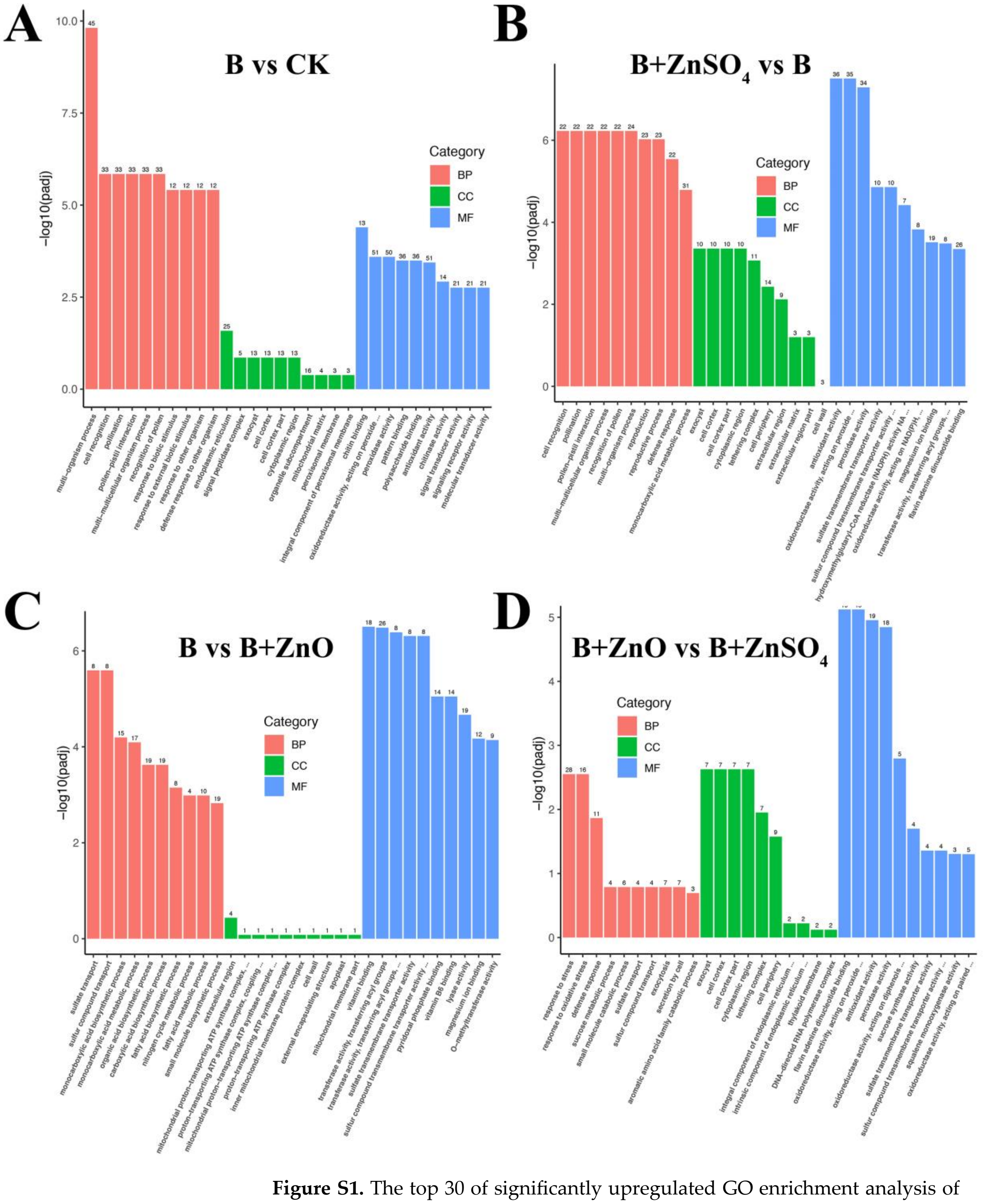

Figure S1. The top 30 of significantly upregulated GO enrichment analysis of differentially expressed genes in (A) B vs CK (B) B+$ZnSO_4$ vs B (C) B+ZnO vs B (D) B+ZnO vs B+$ZnSO_4$.

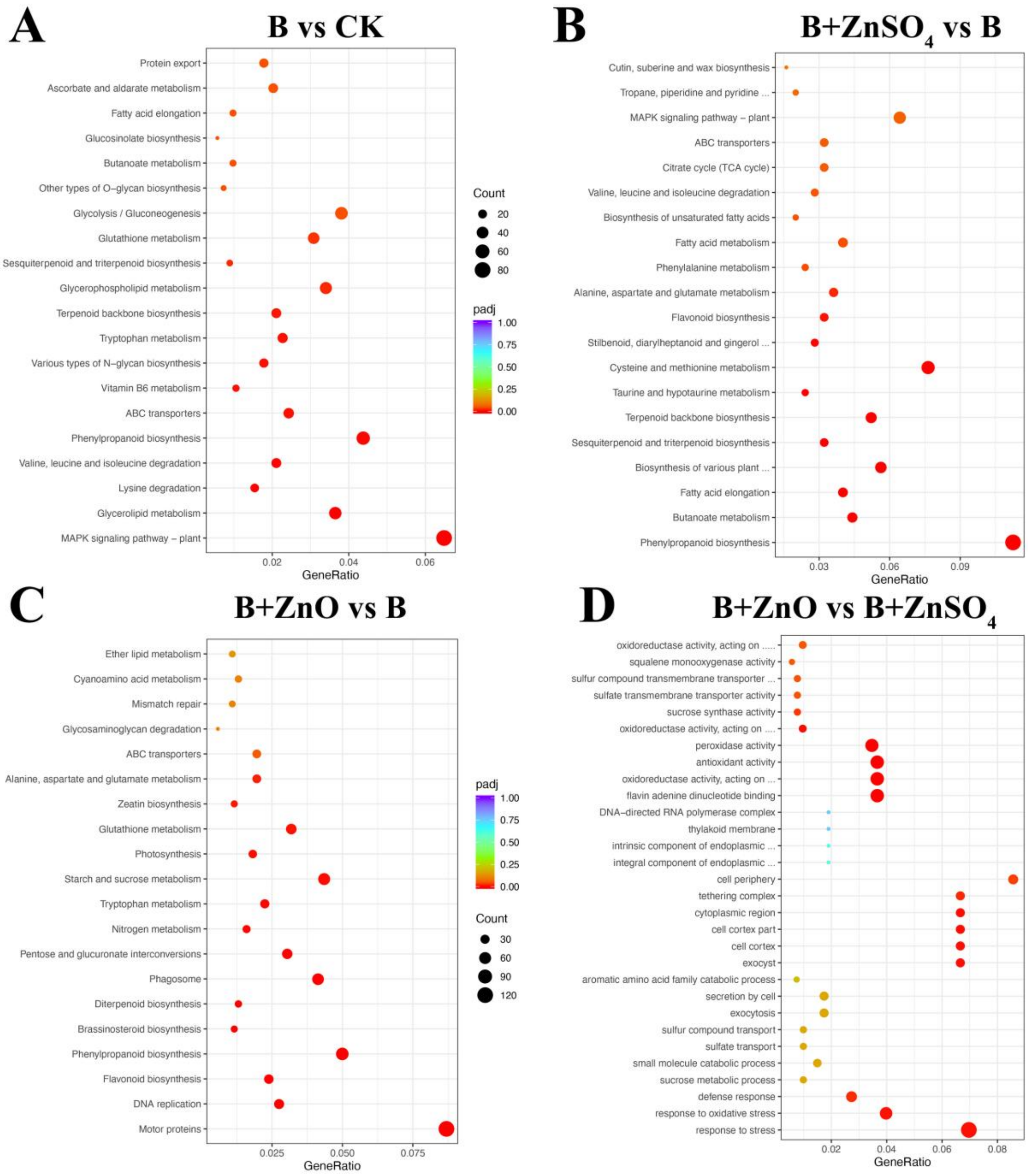

Figure S2. The upregulated KEGG pathway enrichment analysis of differentially expressed genes in (A) B vs CK (B) B+$ZnSO_4$ vs B (C) B+ZnO vs B (D) B+ZnO vs B+$ZnSO_4$.

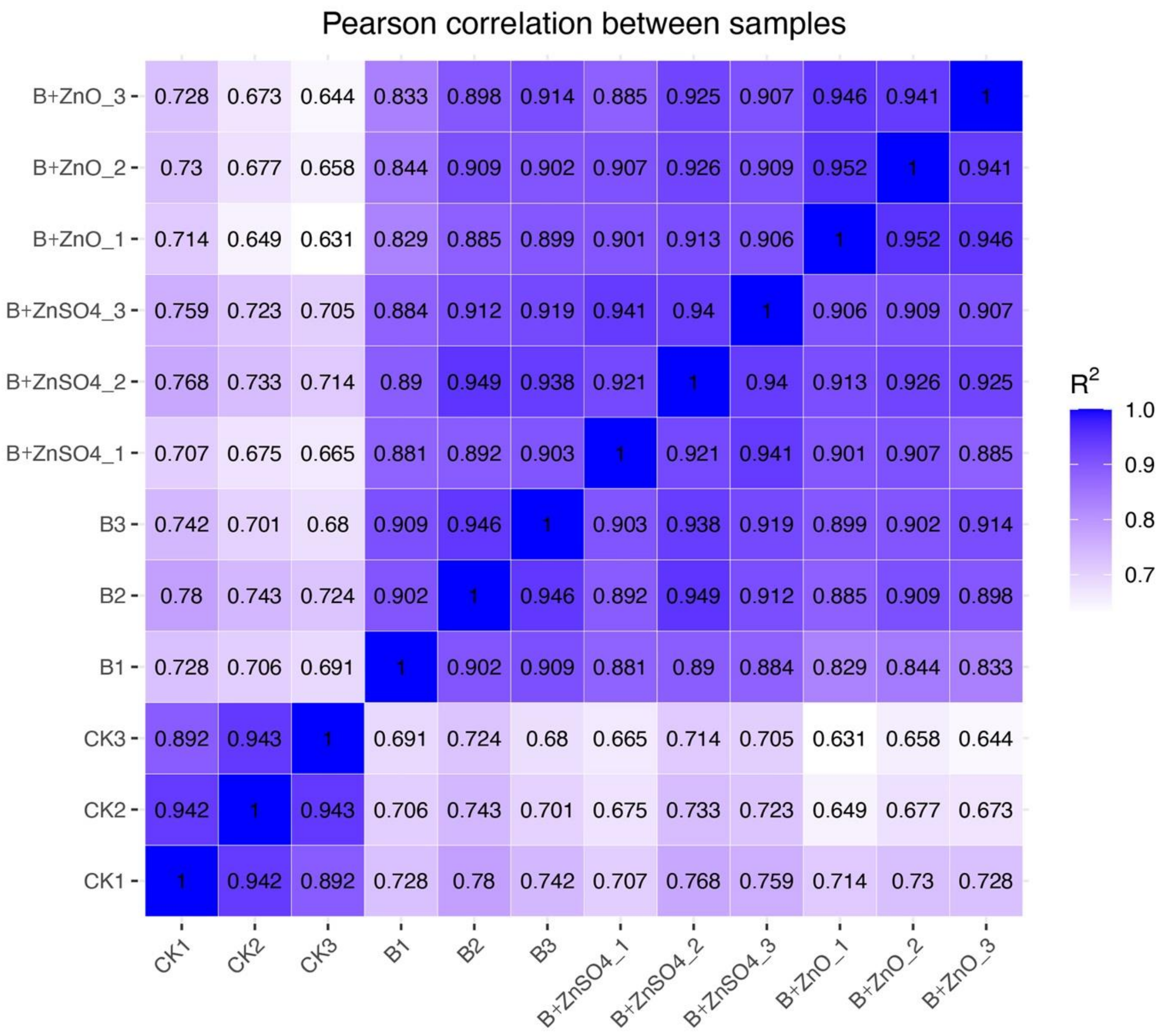

Figure S3. Heat map of inter-sample correlations. The horizontal and vertical coordinates in the figure are the squares of the correlation coefficients of each sample.
